# Potentially inappropriate use of benzodiazepines and z-drugs in the older population—analysis of associations between long-term use and patient-related factors

**DOI:** 10.7717/peerj.4614

**Published:** 2018-05-22

**Authors:** Aliaksandra Mokhar, Niklas Tillenburg, Jörg Dirmaier, Silke Kuhn, Martin Härter, Uwe Verthein

**Affiliations:** 1 Department of Medical Psychology, University Medical Center Hamburg-Eppendorf, Hamburg, Germany; 2 Center for Interdisciplinary Addiction Research, Department of Psychiatry and Psychotherapy, University Medical Center Hamburg-Eppendorf, Hamburg, Germany

**Keywords:** z-drugs, Older people, Psychological and sociodemographic factors, Benzodiazepines, Insomnia, Unemployment, Daily structure, Long-term use

## Abstract

**Introduction:**

The long-term use of benzodiazepines (BZD) and z-drugs in older populations is associated with a variety of sociodemographic and health-related factors. Recent studies reported that long-term BZD and z-drugs use is associated with increased age, female sex, and severe negative psychological (e.g., depression) and somatic (e.g., chronic disease) factors. The current study explores the sociodemographic and health-related factors associated with long-term BZD and z-drugs use in the elderly.

**Methods:**

We conducted a cross-sectional survey among randomly selected patients of one health insurance plan (“AOK North-West”) with BZD and z-drugs prescriptions in the past 12 months. The sample was stratified by appropriate German prescription guidelines (yes vs. no) and age (50–65 vs. >65 years). To examine the association of selected sociodemographic and psychological variables (e.g., sex, employment status, quality of life, depression) with long-term use, a binary logistic regression analysis was conducted.

**Results:**

In total, data from 340 patients were analyzed. The mean age was 72.1 (*SD* = 14.5) years, and the most commonly used substances were zopiclon (38.1%), oxazepam (18.1%), and lorazepam (13.8%). The mean defined daily dose (DDD) was 0.73 (*SD* = 0.47). Insomnia was the main reason for prescribing BZD and z-drugs. The long-term use of BZD and z-drugs was significantly associated with unemployment (*OR* = 2.9, 95% CI [1.2–7.1]) and generally problematic medication use (*OR* = 0.5, 95% CI [0.2–1.0]).

**Discussion:**

Unemployment status and problematic medication use had a significant association with the patient-reported, long-term use of BZD and z-drugs. Divergent prescription patterns might suggest problematic patterns of BZD and z-drugs use. The causal connection between the identified factors and problematic BZD and z-drugs prescription is not discussed in this paper. Nevertheless, employment status and possible evidence of general problematic drug use may be a warning signal to the prescribers of BZD and z-drugs.

## Introduction

Benzodiazepines (BZD) and z-drugs (e.g., zopiclone, zolpidem) are frequently used to treat insomnia and anxiety among elderly patients (Lohse & Müller-Oerlinghausen, 2013; Ashton, 1994). Despite the positive effects of BZD and z-drugs, a variety of negative side effects may occur; these include a risk of falling, cognitive difficulties, abuse and dependence on the drug (Baldwin et al., 2013; Lader, 2011; Cumming & Le Couteur, 2003; Cimolai, 2007). To avoid these negative consequences, international guidelines recommend prescription for acute and short-term treatment only, with “short-term use” not exceeding four weeks; other medical or psychological treatment options should be considered before BZD is chosen (American Psychiatric Association, 1990; Fialova, Topinkova & Gambassi, 2005; Morgenthaler et al., 2007; Joint Formulary Committee, 2010; American Geriatrics Society, 2015). Although the guiding principles of BZD use are clearly defined in clinical practice, long-term use seems to increase with age (Petitjean et al., 2007), leading to a high prevalence rate of long-term users, particularly among the older population (Hogan et al., 2003; Naja et al., 2000; Neutel, 2005; Verthein et al., 2013). A study from Olfson and colleagues shows that the percentage of BZD use increased with age from 2.6% (18–35 years) to 5.4% (36–50 years) to 7.4% (51–64 years) to 8.7% (65–80 years). The proportion of long-term users increased with age from 14.7% (18–35 years) to 31.4% (65–80 years) (Olfson, King & Schoenbaum, 2015). The proportion of German patients using BZD for >6 months increased with age (65–70 years: 12.3%; 71–80 years: 15.5%; 81–90 years: 23.7%; >90 years: 31.6%) (Jacob, Rapp & Kostev, 2017). Recent studies show an increased risk for older women regarding potentially inappropriate use of BZD, indicating a significant difference by sex among older patients (Morgan et al., 2016). Unemployment (Magrini et al., 1996) and female sex (Neutel, 2005; Verthein et al., 2013) are further parameters related to the risks of potentially inadequate BZD or z-drugs intake. In addition to linking patterns of BZD use to demographic, lifestyle and clinical variables, these studies suggest a positive association of long-term BZD use with alcohol consumption, anxiety and psychological stress; exercise was negatively related to chronic use (Nordfjærn et al., 2013). Psychological factors such as anxiety, depression and addiction behavior are associated with problematic misuse use of BZD and z-drugs (Manthey et al., 2012; Kan, Hilberink & Breteler, 2004; Zandstra et al., 2004). In studies looking for association between depressive disorders and the use of BZD, people were more likely to be unemployed, have a history of child abuse, or suffer from comorbid panic disorder and have higher anhedonia scores (Rizvi et al., 2015). When comparing short- and long-term users of BZD, the latter had a more severe history of mental health problems for which they had received more serious treatment, more psychotropic drugs, and more frequent hospital consultations. They were also more likely to suffer from chronical physical illnesses such as diabetes, asthma, chronic obstructive pulmonary disease, hypertension and serious skin disorders (Zandstra et al., 2002). Furthermore, the potentially inappropriate use of especially high doses of BZD and z-drugs reduced quality of life, social functioning, and has high levels of psychological distress (Lugoboni et al., 2014; Tamburin et al., 2017; Ventegodt & Merrick, 2003).

Many factors are associated with long-term BZD and z-drugs use. These patterns suggest that further analysis could reveal important variables that may lead to broader and more evidence-based risk prevention. Avoiding the long-term use of these drugs could be facilitated by the use of risk indicators (Zandstra et al., 2002). These may produce a decline in long-term BZD use, especially in elderly patients. Consequently, there is great need to understand these patterns and determine the patient-related characteristics associated with long-term BZD use. Awareness of these patient-related factors may lead to adjustments in the care offered to this older population. Therefore, this paper sought to analyze the association between patient characteristics (such as sociodemographic factors or psychological and clinical components) and the long-term use of BZD and z-drugs.

## Methods

The study design is a cross-sectional survey of data sampled from people over the age of 50, all of whom were recipients of BZD or z-drugs prescriptions. The study was carried out as part of the project “Benzodiazepines and z-drugs—concepts for risk reduction among older patients”, sponsored by the Federal Ministry of Health. Data were accessed through the German health insurance AOK North-West. Relevant patient data were stratified by age (50–65 vs. >65 years) and by prescription behavior (short-term versus long-term intake). The risk of patients forgetting the reasons for initial use was reduced by limiting the intake time to a maximum of five years. The last prescription of BZD and z-drugs, whether adhering to guidelines or not, must have occurred within the previous twelve months. Stratification by age was used to help uncover specific causes, symptoms and attendant circumstances likely involved with long-term consumption of BZD and z-drugs among elderly patients. The group stratification was as follows: (1) potentially inappropriate or long-term users (<than four weeks) are patients with two currently consecutive or more than two consecutive prescriptions in the past year (2013–2014); (2) potentially appropriate or short-term (>as four weeks) users were patients with only one prescription in the past year (2013–2014) and no prescriptions in the previous year (2012–2013). Ethical approval was obtained from the board of the medical ethics committee of Hamburg (PV4688).

### Procedure

A group size of *N* = 100 was sufficient to substantiate statistically relevant small- to medium-effect sizes with a steady distribution of characteristics (*d* ≥ .30, *α* = 0, 05, Power = 80%) and medium effects with differences of relative frequency (Diff. ≥ 20%, *OR* ≥ 2, 3). AOK North-West has 1.4 million insured persons. German insurance police data suggested that approximately 5% of the insured population received prescriptions of BZD or z-drugs within one year and that at least 16% had long-term prescriptions. Elderly patients are overrepresented in this finding. Thus, 11,000 AOK insured members were eligible for the target group of long-term intake, and the total of short-term users was estimated to be approximately 44,000 persons. Based on experience, AOK predicted a feedback rate of 10%. Based on this, 4 × 1,000 patients were contacted (according to the stratification outlined above). Four thousand eligible people, one thousand per stratum, were coincidentally identified through insurance databases and contacted by their insurance company. Over the last several years in Germany, one can observe a trend that may seem at first paradoxical: while the rates of long-term BZD use through government insurance declined, the rates of private prescriptions (in the case of legally insured persons) increased (Hoffmann, Glaeske & Scharffetter, 2006). Thus, it cannot be assumed that the prescriptions have been fundamentally reduced but rather that a shift in the regulations from cash to private prescriptions has occurred. Pharmacists estimate that approximately half of all z-drugs in the western federal states are private prescriptions (Hoffmann, Glaeske & Scharffetter, 2006). Patients who received BZD and z-drugs via private prescription were also included to consider another group potentially affected by long-term use. For this purpose, a Hamburg pharmacist, in the pharmacy proper, asked these patients to participate; another 100 persons were contacted. A personalized and mailed letter invited them to participate in the study. Those who were contacted were asked to complete the questionnaire and to use the anonymous, stamped return envelope, which was sent to the University Medical Center Hamburg-Eppendorf (UKE) research group. To ensure anonymity, participants were reminded throughout not to include any personal information. The respondents did not receive any incentives to participate in the study. The questionnaire survey was run in between December 2014 and February 2015.

### Measures

Sociodemographic data included sex, age, family, living and employment status, care dependence, and questions related to BZD and z-drugs. The analysis of psychological factors was based on the following standardized inventory: anxiety via Generalized Anxiety Disorder questionnaire (GAD-7) (Williams, 2014), depression via Patient Health Questionnaire (Lowe et al., 2004; Kroenke & Spitzer, 2002), alcohol use via AUDIT (Bradley et al., 2007; Daeppen et al., 2000), quality of sleep via Pittsburgh Sleep quality Index (PSQI-short) (Buysse et al., 1989; Carpenter & Andrykowski, 1998), beliefs-related medication use via Beliefs about Medicines Questionnaire (BMQ) (Horne, Weinman & Hankins, 1999; Mahler et al., 2012), health-related quality of life via HRQL-SF-12 questionnaire (Cheak-Zamora, Wyrwich & McBride, 2009) and general medication use via a short questionnaire of medication use (Watzl et al., 1991) (German: Kurzfragebogen für Medikamentengebrauch, KFM). The short survey for drug use (KFM) is a validated and standardized 12-item survey used to assess the abuse of sleep-inducing drugs, mood stabilizers and tranquilizing medications. It is often used in research and epidemiological studies on drug use in Germany (Watzl et al., 1991). The BMQ Specific comprises two factors assessing beliefs about the necessity of prescribed medication (Specific-Necessity) and concerns about prescribed medication based on beliefs about the danger of dependence and long-term toxicity and the disruptive effects of medication (Specific-Concern).

### Statistical analyses

Descriptive statistics were calculated to present demographic characteristics. Adjustment for missing values was carried out by replacing the value (without imputation), which did not occur below the specified values. To analyze differences between long- and short-term users, we used *χ*^2^ tests for categorical variables and *t*-tests for metric variables. To reduce multicollinearity, we used univariate analysis (*R*, Person-Product-moment correlation coefficient) to examine highly correlated variables and remove one of the pair for the final model. For effect sizes, Cohen’s d was calculated with the effect size calculator of the University of Colorado (Becker, 1998). Finally, to examine the association of the selected sociodemographic and psychological variables for long-term use, binary logistic regression analysis was conducted. We used the forward entry method to test all variables in one model. To analyze the variables of this method for content, the threshold values of categorical scale were dichotomized. Nagelkerke’s *R*^2^ and the Hosmer-Lemeshow statistic were calculated as a goodness-of-fit measure for the model. All analyses were performed using Statistical Package for the Social Sciences (SPSS).

## Results

As outlined above, 4,000 AOK patients were contacted (based on the stratification categories of age and appropriate prescription) and asked to fill out and return a nine-page questionnaire. A total of 466 persons completed the questionnaire, which produced a feedback rate of 11.7%. The response rate was considered to be 12%. Following a preliminary discussion with AKO NordWest, corresponding experiences should only be provided with a certain degree of process depth. In addition, 16 participating pharmacists were able to procure 43 private patients, falling short of the target number of 100. Overall, there were 509 questionnaires completed, and the projected recruitment target of 466 patients was nearly reached. However, only 340 persons were included in the statistical analyses, as 169 individuals, mainly from the group of short-term users, did not record their intake of benzodiazepines or z-drugs. The extent to which missing answers could be attributed to memory lapses, ignorance about the medication or actual non-use of these prescription medications cannot be assessed. Most of the 340 patients were married, lived with partners/children, were unemployed and were not care dependent. The most commonly used substances were zopiclon, oxazepam, and lorazepam (Table 1). The defined daily dose (DDD) was 0.73 on average. The standardized daily dose (DDD) is statistically significantly higher than those with guideline-based prescription rate. In both patients group was the medications taken in a lower dose, on average less than one DDD.

**Table 1:** Patient characteristics with medication intake according to prescription type.

Characteristic	Appropriate users	Inappropriate users	Total	Test (*df*)	*p*	d/V
Sex				*χ*^2^ (1)	0.991	0.00
Female	68.5%	68.5%	68.5%			
Male	31.5%	31.5%	31.5%			
Age, M (*SD*)	68.4 (12.7)	73.7 (15.0)	72.1 (14.5)^**^	*t*-test (303)	0.004	0.37
Marital status			^*^	*χ*^2^ (1)	0.027	0.19
Unmarried	49.5%	62.4%	58.6%			
Married	50.5%	37.6%	51.4%			
Living situation				*χ*^2^ (2)	0.919	0.02
With children/partner	54.0%	53.0%	53.3%			
Alone	36.0%	35.5%	35.6%			
Retirement home	10.0%	11.5%	11.1%			
Employment			^**^	*χ*^2^ (1)	0.004	0.16
Unemployed	73.5%	86.6%	82.6%			
Employed	26.5%	13.4%	17.4%			
Care dependency				*χ*^2^ (1)	0.060	0.10
Yes	19.4%	29.4%	26.4%			
No	80.6%	70.6%	73.6%			
Primary medication			^***^	*χ*^2^ (17)	0.000	0.00
Zopiclon	29.2%	42.0%	38.1%			
Oxazepam	20.8%	17.0%	18.1%			
Lorazepam	18.8%	11.6%	13.8%			
Zolpidem	6.3%	11.2%	9.7%			
Diazepam	11.5%	3.6%	5.9%			
Bromazepam	8.3%	1.3%	3.4%			
Other	5.2%	13.4%	10.9%			
Defined daily dose DDD, M (*SD*)	0.62 (0.40)	0.76 (0.48)	0.73 (0.47)^*^	*t*-test (212)	0.034	0.31
*N*	102	238	340			

**Notes.**

Abbreviations *N* number *df* degrees of freedom *d* effect size *M* mean *SD* standard deviation *p* significance *T*- and *χ*^2^-Test

* *p* < 0.05.

** *p* < 0.01.

*** *p* < 0.001.

The univariate analysis (Table 1) looks at substantial differences between short-term and long-term users of the medication. It shows that long-term users were significantly older than short-term users. While short-term users were more likely to be married or in a relationship, long-term users were more often divorced or widowed. Furthermore, long-term users were more often divorced or widowed. Furthermore, long-term users were more likely to be unemployed than short-term users were. Short-term users of BZD and z-drugs needed care less often than long-term users did. Finally, long-term users consumed a greater amount z-drugs, while more short-term users took BZD and z-drugs; the DDD is significantly higher among long-term users.

In the next methodic step the univariate analysis was carried out (Table 2). The univariate analysis was conducted to determine the correlations between the analyzed psychological variables. The mean average of PHQ-9 for surveying depression, as well as that of GAD-7 for measuring anxiety, suggest a light to moderate manifestation of the respective symptoms in each group, without a significant difference between the groups. The screening instrument AUDIT-C, which investigates the risks of incurring alcohol-related dysfunctions, suggests a low rate on average in both groups. SF-12 evaluation of health-related quality of life regarding physical state indicates below average statistics for both groups; data in this field are even lower than those concerning mental well-being. Figures from the Pittsburgh sleep quality index suggest poor sleep quality among both groups. The BMQ necessity spectrum, meanwhile, produces a mean average of *M* = 3.3 (*SD* = 1.14) and suggests a significant difference between the groups. This strongly indicates that patients consider BZD and z-drugs intake vital, particularly those belonging to the group of long-term users. In contrast, the chart looking at concerns regarding BZD and z-drugs intake generates a low mean average, which suggests indiscriminate use of the medication. The KFM-Score examined problematically medication intake indicates a differences in the groups to show the significantly accordance between long term users and problematically medication use score.

**Table 2:** Univariate analysis of analyzed variables by prescription type.

Variables	Appropriate users	Inappropriate users	Total	*df*	*p*	*d*
GAD-7-score	0,56 (0,87)	0,73 (0,95)	0,68 (0,93)	296	0,164	0,19
PHQ-9-score	1,35 (1,19)	1,34 (1,23)	1,35 (1,22)	331	0,765	0,01
Audit-C-score	1,80 (0,40)	1,81 (0,39)	1,81 (0,39)	328	0,507	0,03
PSQI-score; M (*SD*)	1,73 (0,73)	1,70 (0,72)	1,71 (0,72)	332	0,872	0,04
KFM-score	1,46 (0,50)	1,60 (0,49)	1,56 (0,50)	327	0,012^*^	0,28
BMQ-N-score; M (*SD*)	3,06 (1,24)	3,41 (1,08)	3,31 (1,14)	304	0,008^**^	0,30
BMQ-C-score; M (*SD*)	2,48 (1,13)	2,89 (0,99)	2,55 (1,03)	271	0,145	0,39
SF12-P-score; M (*SD*)	35,96 (11,89)	36,60 (9,60)	36,40 (10,40)	330	0,610	0,06
SF12-M-score; M (*SD*)	41,62 (11,73)	42,87 (10,03)	42,49 (10,57)	330	0,320	0,11
*N*	102	238	340			

**Notes.**

Abbreviations *N* number *df* degrees of freedom *d* effect size *M* mean *SD* standard deviation *p* significance *T*- and *χ*^2^-Test PSQI Pittsburgh Sleep quality Index PHQ-9 Patient Health Questionnaire GAD-7 Generalized Anxiety Disorder questionnaire AUDIT-I Alcohol Use Disorders Identification Test Consumption BMQ Beliefs about Medicines Questionnaire specific N necessity C concerns KFM short questionnaire of medication use SF12 short version of health related quality of life (HRQL) *P* physical *M* mental

* *p* < 0.05.

** *p* < 0.01.

*** *p* < 0.001.

Table 3 presents an analysis of multi-collinearities through the partial correlations between the variables examined. There is no chance of multi-collinearity if the correlation coefficients are smaller than the value of 0.8 (Schendera, 2014). Overall, no strong correlations of variables were detected. The manifestations of PHQ-9 and GAD-7 show moderate positive correlation, which is comprehensible on a symptom level. Both disorders are frequently co-morbid. Moderately strong negative correlations can be seen between SF-12 mental scale and GAD-7 as well as PHQ-9. This interrelation is understandable, since strong mental quality of life is incompatible with anxiety and depression. Another negative correlation can be found between SF-12 physical scale and need of care. Poor physical quality of life and need of care are comprehensible. Finally, it becomes apparent that people at an advanced age are more likely to be married or in a relationship, as well as working less or retired.

**Table 3:** Pairwise correlations between analyzed variables.

	**Age**	**Sex**	**MS**	**LS**	**ES**	**CD**	**GAD-7**	**PHQ-9**	**Audit-C**	**KFM**	**PSQI**	**BMQ-N**	**BMQ-C**	**SF12-M**	**SF12-P**
**Age**	1														
**Sex**	−0,238^***^	1													
**MS**	−0,263^***^	−0,272^**^	1												
**LS**	0,065	−0,075	−0,571^**^	1											
**ES**	0,429^***^	0,054	−0,171^*^	−0,118^***^	1										
**CD**	0,295^***^	−0,087	−0,120^*^	−0,076	0,272^***^	1									
**GAD-7**	−0,320^**^	0,094	−0,070^*^	−0,018	0,111^*^	0,021	1								
**PHQ-9**	−0,165^***^	0,022	−0,022	0,052	−0,011	−0,093	0,600^***^	1							
**Audit-C**	−0,274^***^	0,24^***^	−0,215^**^	0,181^***^	0,229^***^	0,219^***^	0,034	0,108	1						
**KFM**	−0,041	−0,080	−0,120	0,138	−0,055	−0,057	0,267^**^	0,401^***^	0,143^*^	1					
**PSQI**	−0,005^*^	−0,005	−0,070	0,035	−0,084	−0,108	0,436^***^	0,709^***^	0,144^*^	0,346^***^	1				
**BMQ-N**	−0,081	0,069	−0,064	−0,089	−0,269^*^	−0,169^**^	0,204^***^	0,279^***^	0,218^***^	0,383^***^	0,288^***^	1			
**BMQ-C**	−0,149^*^	0,079	0,112	0,035	−0,118^*^	0,001	0,346^***^	0,386^**^	0,087	0,319^***^	0,326^***^	0,447^***^	1		
**SF12-M**	−0,200^*^	−0,050	−0,036	0,002	−0,052	0,029	−0,510^***^	−0,622^***^	0,009	−0,345^***^	−0,491^***^	−0,282^***^	−0,489^***^	1	
**SF12-P**	−0,467^***^	0,118^*^	0,149^*^	−0,055	0,345^***^	0,491^***^	0,014	−0,147^*^	−0,280^***^	−0,227^***^	−0,315^***^	−0,182^***^	−0,090	0,017	1

**Notes.**

* *p* < 0.05.

** *p* < 0.01.

*** *p* < 0.001.

Abbreviations MS marital status LS living situation ES employment status CD care dependency PSQI Pittsburgh Sleep quality Index PHQ-9 Patient Health Questionnaire GAD-7 Generalized Anxiety Disorder questionnaire AUDIT-I Alcohol Use Disorders Identification Test Consumption BMQ Beliefs about Medicines Questionnaire specific N necessity C concerns KFM short questionnaire of medication use SF12 short version of health related quality of life (HRQL) *M* mental *P* physical

Table 4 presents results from binary logistic model examining the association between potentially inappropriate as a long-term use and sociodemographic as well as psychological variables. For measuring depression and anxiety values as predictors were selected, ranging from zero to mild symptomatology. This was done because the sample analyzed was not clinical and did not show any distinct symptomatology. The model shows that unemployment (*OR* = 2.9, *p* = 0.021) and problematically medication use (*OR* = 4.7, *p* = 0.055) are statistically significantly associated with long-term use. All other of the analyzed variables were not significantly associated. The total explained variance amounted to 24.1% and the Hosmer-Lemeshow statistic indicates a good fit (Horne, Weinman & Hankins, 1999).

**Table 4:** Associations of potentially inappropriate use of BZD and z-drugs and patient related factors.

Predictors	OR	95% CI		*p*
Female sex	0.648	0.303	1.386	0.264
Higher age	1.023	0.988	1.060	0.193
Unmarried	2.440	0.905	6.580	0.078
Living alone	2.620	0.707	9.707	0.149
Unemployment	2.898	1.176	7.141	0.021^*^
Care dependency	1.819	0.651	5.085	0.254
Mild anxiety (GAD-7-score)	0.404	0.086	1.891	0.250
Minor depression (PHQ-9-score)	7.208	1.425	0.282	0.668
Problematically alcohol use (Audit-C-score)	1.579	0.653	3.820	0.311
Problematically medication use (KFM-score)	4.741	2.161	0.985	0.055^*^
Problematically sleep (PSQI-score)	0.928	0.459	1.876	0.835
Necessity of BZD (BMQ-N-score)	1.259	0.714	1.579	0.204
Concerns about BZD (BMQ-C-score)	1.061	0.714	1.579	0.768
Mental quality of life (SF12-M-score)	1.036	0.991	1.083	0.117
Physical quality of life (SF12-P-score)	1.033	0.992	1.075	0.120

**Notes.**

Nagelkerke’s *R*^2^ = 0.241

Hosmer-Lemeshow Goodness-of-Fit Test *χ*^2^ = 10.250, *p* = 0.248

* *p* < 0.05.

** *p* < 0.01.

*** *p* < 0.001.

Abbreviations OR odds ratio PSQI Pittsburgh Sleep quality Index PHQ-9 Patient Health Questionnaire GAD-7 Generalized Anxiety Disorder questionnaire AUDIT-I Alcohol Use Disorders Identification Test Consumption BMQ Beliefs about Medicines Questionnaire specific N necessity C concerns KFM short questionnaire of medication use SF12 short version of health related quality of life (HRQL) *P* physical *M* mental

## Discussion

In the univariate analysis we found that long term users were older, were more likely to be unmarried and unemployed and used a different primary medication compared with short term-users. Regarding the psychological variables, long-term users had significantly higher scores on BMQ necessity and KFM than short-term users did. The multivariate analysis identified unemployment status and problematic medication use as clear factors of long-term use. All other analyzed independent variables were not significant for a reliable association.

This study did not identify either sex or age as predictors for long-term intake of BZD and z-drugs. This partly contradicts the results of epidemiological studies (Verthein et al., 2013) that analyzed benzodiazepine prescriptions over a prospective period of 12 months within a larger sample consisting of patients of all ages. It can be argued that age is a predictor of long-term use when comparing younger adults with the elderly, but within the group of people over 50 years, age does not seem to influence use. The present research focused on people age 50 and older and was not epidemiological in nature. Sex as a predictor could not be confirmed in the present study. As in previous research (Verthein et al., 2013; Kan, Hilberink & Breteler, 2004), there were more female users (68.5%) than males in the sample, but there were no differences in their tendencies to use the medication in a long-term manner. Marital and living-situation status were not predictors for long-term intake in our analysis. Long-term intake, as established by KFM, reveals a difference between the groups examined and may serve as a predictor. The questionnaire increased the stratified breakup of the groups. It becomes clear that older people who use BZD and z-drugs for more than four weeks may continue their problematic consumption (not just regarding the time criterion). The attitudes (BMQ) toward medication use reveal that patients consider BZD intake to be vital, a view that is especially prevalent among long-term users of the drug. Meanwhile, the chart looking at concerns about drug consumption produced a low mean average, which allows for the view that indiscriminate use of BZD or z-drugs is widespread. The idea that divergent prescription harbors problematic patterns of drug use, questioning previous treatment methods, is beyond dispute at this point.

The survey at hand used PSQI to collect data about this condition; not surprisingly, both groups—all older patients with short-term intake and those without it—seem to experience sleeping difficulties. This result is consistent with findings from other studies (Poyares et al., 2004; Vaapio et al., 2015; Beland et al., 2010). People are likely to experience sleep disorders, regardless of the length of BZD and z-drugs intake. Earlier studies revealed that nearly every elderly patient reported having at least one symptom of insomnia, while those with medical problems were particularly likely to suffer from symptoms (Foley et al., 2004; Foley et al., 1995). The long-term use of BZD and z-drugs for insomnia seems to be an effective of treating symptoms. The result, however, can be low-dose addiction, which constitutes a new problem. At this point, the benefits of ongoing short-term treatment of symptoms should be weighed against its consequences, namely, drug dependency. The respective pros and cons of treatment should be appraised. Alternative medicinal and non-medicinal treatment options should also be considered. Both doctors and patients should engage in a decision-making process to achieve a shared ideal outcome. Established approaches regarding topics such as medicinal over-prescription are applicable to this field.

Unlike other studies (Manthey et al., 2012; Kan, Hilberink & Breteler, 2004; Morin, Belanger & Bernier, 2004) depression and anxiety were not confirmed as predictors. This survey uses the value “mild depression” when examining patients already suffering from depressive symptoms. It emerges that mild depressive symptomatology increases the likelihood of long-term intake, contradicting studies looking at the correlation between depression and BZD and z-drugs use. These studies focused on specific facets of depression (e.g., diagnosed depression, heavy episodes, comorbid disorder) in combination with equally specific forms of BZD intake (BZD and z-drug addiction). As with depression, the value “mild anxiety” was used for symptoms of anxiety. We assumed that patients taking BZD and z-drugs temporarily to combat a state of anxiety would discontinue the drug after a short while, resulting in fewer symptoms. Patients with long-term intake, however, will continue to deal with symptoms of anxiety. Still, we could not identify mild anxiety as a predictor.

Our results show that unemployment is a significant factor for the long-term use of BZD and z-drugs. It is known that people without employment, daily structure, social contacts and daily goals have a higher risk of developing a mental disorder (Butterworth et al., 2012; Perreault et al., 2016). It is also known that problematic substance use increases the likelihood of unemployment and decreases the chance of finding and holding down a job. Conversely, it can be assumed that people use the medication over the long term because they are unable to work for health reasons (Henkel, 2011). Research shows that unemployment, on average, has a negative impact on an individual’s psychological and physical well-being (McKee-Ryan et al., 2005). Knowing this, particularly in the case of the elderly, suggests that attention should be paid to employment and, after the pension, to everyday life activity to maintain a daily structure and social contacts and thereby improve or maintain their well-being and reduce the risk of long-term medication use.

In the present analysis, the quality of life as a parameter does not differ between the groups and, thus, cannot serve as predictor for long-term consumption. This outcome runs counter to previous research (Gelatti et al., 2006; Gonzalez-Salvador et al., 2000). Both groups, however, show that mental well-being is considered more important than physical fitness with increased age. The greatest discomfort was observed in items exploring physical ability. The long-term use of BZD and z-drugs affects this area the most, in accordance with previous research (Gray et al., 2006). As elderly people are more susceptible to physical infirmity, this result is understandable (Gnjidic et al., 2009; Berdot et al., 2009).

## Conclusion

As no psychological predictors emerge from our analysis, a closer look is needed at the daily routine of older adults, at their employment status, and at their medication use overall. Long-term consumption, perhaps, can be explained but not yet predicted by anxiety, depression, and sleep disorders or poor quality of life. At this point, our results point away from symptoms: daily activities and routine, purposeful performance of a task, and engaging with other people all contribute significantly to the non-problematic consumption of BZD and z-drugs. Further research and clinical discussion should continue from here to find solutions of a systemic nature.

## Limitations

Cross-sectional studies cannot conduct causal tests. The results of this paper should therefore be interpreted with caution. Furthermore, the results of this stratified study do not lend themselves to generalization. They merely provide reference points. Further longitudinal studies should be carried out to address the question of predictive values regarding potentially inappropriate intake of sedatives and hypnotics. Many important factors such as physical well-being, chronic ailments, and additional medication use were not analyzed in this study. Attempts to discontinue the drug were not considered, and the role of the prescriber was excluded.

## Supplemental Information

10.7717/peerj.4614/supp-1 Supplemental Information 1 Raw data for the final analysis

10.7717/peerj.4614/supp-2 Supplemental Information 2 Final syntax of the analysis

## Additional Information and Declarations

### Data Availability

The following information was supplied regarding data availability: The raw data are provided in Supplemental Files.
